# Overexpression of Ubiquitin-Conjugating Enzyme E2C Is Associated with Worsened Prognosis in Prostate Cancer

**DOI:** 10.3390/ijms232213873

**Published:** 2022-11-10

**Authors:** Xiaobo Wu, Xingbo Long, Chenkai Ma, Yin Celeste Cheuk, Mengbo Hu, Jimeng Hu, Haowen Jiang

**Affiliations:** 1 Department of Urology, Huashan Hospital, Fudan University, Shanghai 200031, China; 2 Department of Urology, Sun Yat-sen University Cancer Center, Guangzhou 510060, China; 3 Diagnostic Solution, Nutrition and Health, CSIRO Health and Biosecurity, Black Mountain, Canberra, ACT 2601, Australia

**Keywords:** prostate cancer, UBE2C, TP53, biomarkers

## Abstract

To evaluate the role of ubiquitin-conjugating enzyme E2C (UBE2C) in prostate cancer (PCa) progression and prognosis, the TCGA and our PCa tissue microarray cohort were included in the study. Weighted gene co-expression network analysis (WGCNA) and non-negative matrix factorization were used to cluster patients and to screen genes that play a vital role in PCa progression (hub gene). Immunohistochemistry staining was used to evaluate the protein level of UBE2C in prostatic tissues. Through WGCNA, we found a gene co-expression module (named the purple module) that is strongly associated with the Gleason score, pathologic T stage, and biochemical recurrent status. Genes in the purple module are enriched in cell cycle and *P53* signaling and help us to cluster patients into two groups with distinctive biochemical recurrent survival rates and *TP53* mutation statuses. Further analysis showed UBE2C served as a hub gene in the purple module. The expression of UBE2C in PCa was significantly higher than that in paracancerous tissues and was remarkably associated with pathologic grade, Gleason score, and prognosis in PCa patients. To conclude, UBE2C is a PCa-progress-related gene and a biomarker for PCa patients. Therapy targeting UBE2C may serve as a promising treatment of PCa in the future.

## 1. Introduction

Prostate cancer (PCa) remains the most frequently diagnosed solid organ cancer in men, ranked first in new cases and second in deaths among men with cancer in the U.S. [1]. Metastasis causes more than 90% of cancer-related deaths, and most PCa patients also die from metastasis [2]. The growth of malignant prostate tissue is regulated by androgen via the activation of the androgen receptor (AR). Thus, the main therapy for metastatic PCa (mPCa) is androgen-deprivation therapy (ADT), which provides temporary control of the disease. Unfortunately, PCa cells eventually become castration-resistant, which causes tumor progression to metastatic castration-resistant prostate cancer (mCRPC).

Ubiquitin-conjugating enzyme 2C (UBE2C) is essential to the ubiquitin-conjugating enzyme complex. Studies have demonstrated that the dysregulation of UBE2C tends to be positively correlated with the occurrence and progression of solid cancer, and inhibition against UBE2C has been considered a novel therapeutic target for solid cancer [3,4,5]. Wang et al. found that AR-independent PCa is highly related to UBE2C [6]. In AR-independent PCa, epigenetic markers were recruited in UBE2C enhancer areas and activated UBE2C. UBE2C would be a biomarker for PCa as it is crucial to identify the androgen-independent PCa and response to castration therapy [7]. Wang et al.’s study also showed that UBE2C activates cell cycle G2-M progression, and the inhibition of AR-related coactivators could attenuate the activation of UBE2C and induce cell cycle arrest [8].

No evidence supports the prognostic significance of UBE2C in PCa. Moreover, the molecular mechanism and specific role of UBE2C expression in PCa remain unclear. Herein, using data from our cohort and public cohorts, we explore the possible role of UBE2C in PCa.

## 2. Results

### 2.1. Screening PCa Progression-Related Hub Genes

**Figure 1:**
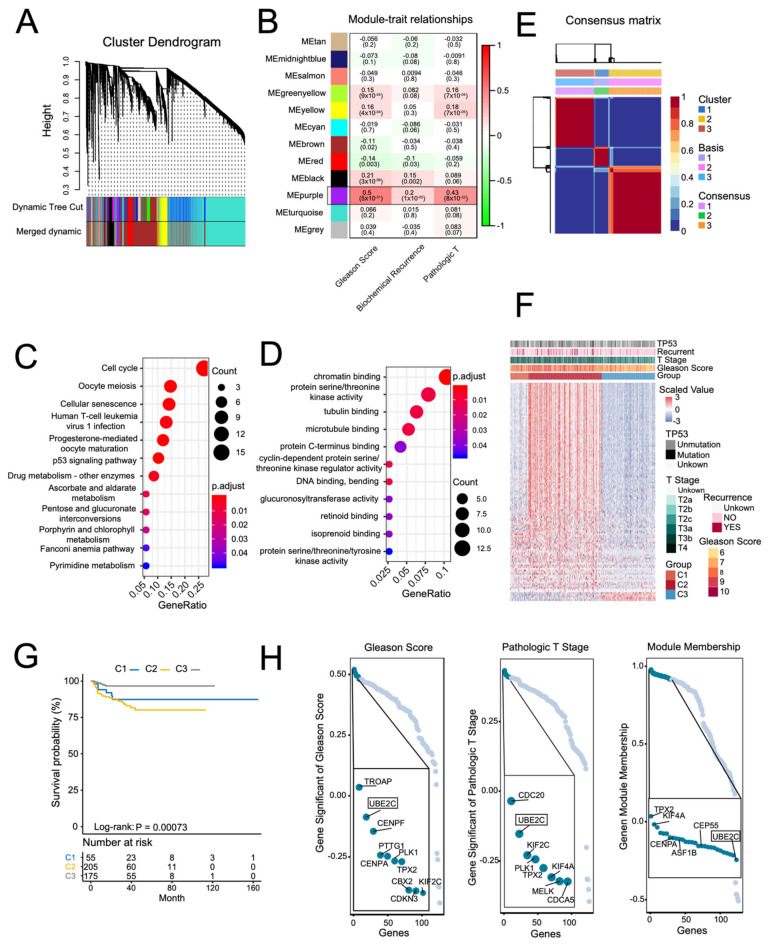
Screening PCa-progress-related hub genes in the TCGA PCa cohort using weighted gene co-expression network analysis (WGCNA). (**A**) A dendrogram generated using the WGCNA, which identified 17 distinct gene modules. Each module is assigned a unique color. (**B**) Pearson’s correlation coefficient (PCC) matrix between module eigengenes (M.E.s) and clinical traits. The PCC values range from −1 to 1 depending on the strength of the relationship. A positive value indicates that the genes within a particular co-expression module increase as the clinical trait increases, whereas the opposite is true if the PCC is negative. Each PCC value is accompanied by the corresponding *p*-value in brackets. C and D: representative enriched G.O. functions (**C**) and KEGG pathways (**D**) of genes in the purple module. (**E**) Non-negative matrix factorization (NMF) using the genes in the purple module divided the TCGA PCa cohort into three distinct clusters. (**F**) Heatmap of the expression of genes in the purple module, clinical features, and *TP53* mutation across the three NMF clusters. (**G**) Kaplan–Meier curve of PSA recurrence-free survival (RFS) rates in different NMF clusters. H: representative genes with high gene significance of Gleason score, pathologic T, and module membership in the purple module. (**H**) Rank for genes in purple module based on gene significance for Gleason Score (Left) and pathologic T stage (Middle), and gene module membership (Right).

To explore hub genes in the PCa progression and invasion, WGCNA used the top 8269 variation genes (the top 8269 genes with the largest variance between samples) in the TCGA PCa cohort to compile the co-expression network. Adhering to the scale-free topology criterion, β = 16 was considered in this study. Following dynamic tree cutting, the topological overlap clustering dendrogram identified 17 distinct gene modules (Figure 1A). The gray module consisted of genes that did not group into any specific module. To identify co-expression modules associated with sample traits (Gleason scores, pathologic T stage, and biochemical recurrent status), we assessed the relationship of the above three sample traits with the module eigengene. Figure 1B shows that the purple module has the strongest association with Gleason scores (0.5, *p* < 0.001), pathologic T stage (0.43, *p* < 0.001), and biochemical recurrent status (0.2, *p* < 0.001). Therefore, we focused on the purple module. Not surprisingly, G.O. and KEGG enrichment analyses showed that genes in the purple module were enriched in the cell cycle, *P53* signaling pathway, DNA binding, bending functions, and pathways (Figure 1C,D).

By using the genes in the purple module and NMF analysis, we divided the PCa patients into three distinct clusters. Figure 1F shows that the genes in the purple module were enriched in the C2 cluster. Additionally, patients in the C2 cluster had higher Gleason scores, pathologic T stages, and *TP53* mutations. Moreover, Kaplan–Meier analysis showed that the C2 cluster had significantly worse PSA recurrence-free survival (RFS) rates (Figure 1G). Finally, we identified hub genes in the purple module based on scores: (1) module membership (high-connectivity genes in the module); (2) gene significance for Gleason score and pathologic T stage. After screening, we found that the UBE2C gene with high module membership in the purple module had higher gene significance scores for both Gleason score and pathologic T stage (Figure 1H). Supplementary Figure S1 shows the co-expression networks of the top 600 ranked gene connection weights in the purple module. We found that UBE2C presented among high degree genes in the co-expression networks. Additionally, previous studies have demonstrated that UBE2C regulated the cell cycle in PCa cell lines in vitro [7]. These results indicate that UBE2C may play an essential role in the purple module co-expression network and is highly associated with PCa progression and invasion.

### 2.2. UBE2C Is Strongly Associated with the Malignant Level of PCa

**Figure 2:**
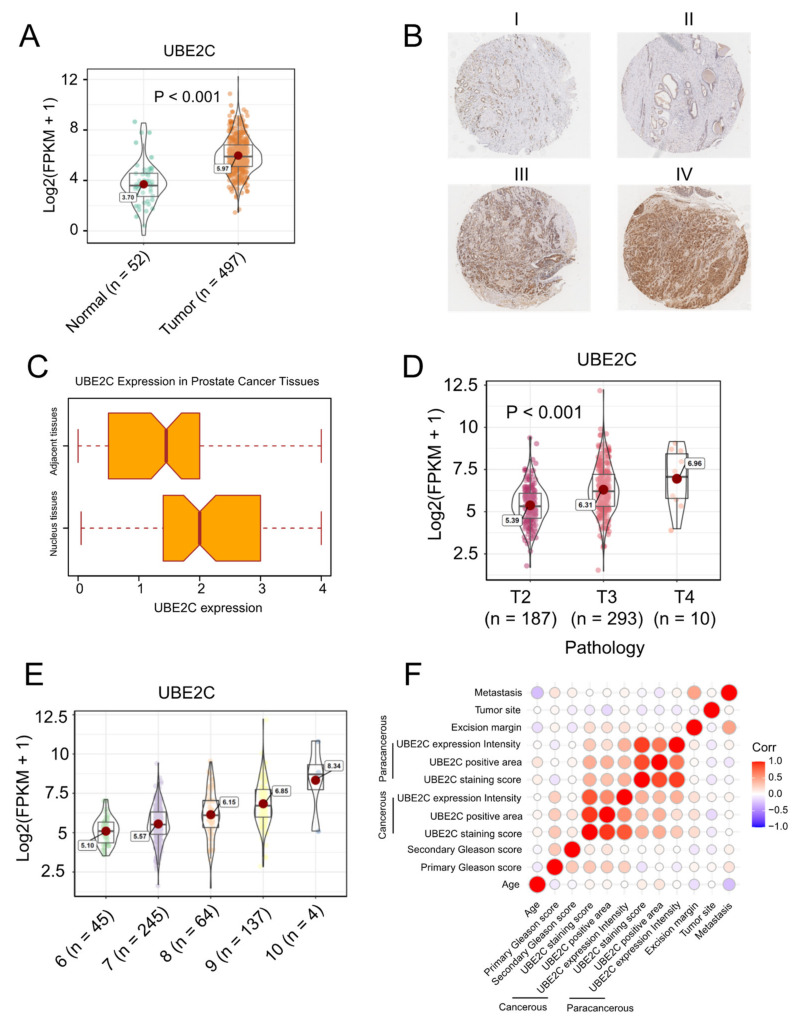
UBE2C is strongly associated with a malignant level of PCa in both TCGA and our cohort. (**A**) The expression of UBE2C in PCa was significantly higher than that in normal prostate tissue. (**B**) The represented pictures of UBE2C protein expression in the prostatic tissues (magnification scale 100×) I. IHC score 4 = 100% × 4 II. IHC score 4 = 100% × 4 III. IHC score 0.1 = 10% × 1 IV. IHC score 0.7 = 70% × 1. (**C**) The immunohistochemical characteristics of UBE2C expression in PCa tissues and the adjacent tissues (paired Wilcoxon test, *p*-value = 0.0002446). (**D**) In the TCGA cohort, we observed the highest UBE2C levels in the T4 stage of PCa. (**E**) The levels of UBE2C also increased as the Gleason scores increased in PCa. (**F**) Spearman correlation among clinical characteristics of PCa patients and UBE2C protein expression. Tumor Site 0 means bilateral, and 1 means unilateral cancerous cell infiltration. Metastasis denotes whether the metastasis is found in lymph capillaries, capsula prostatica, seminal vesicle, or nerve. Intensity denotes the detected UBE2C expression on the microarray, area is the detected area on the microarray, and expression is the multiple of these two. These results demonstrate that the expression level of UBE2C is strongly associated with PCa malignancy and UBE2C is likely to be a biomarker for PCa.

To test whether UBE2C would be a biomarker in PCa, we investigated the expression levels of UBE2C in PCa and normal prostate tissues. A total of 497 PCa samples and 52 benign prostate tissue samples from the TCGA dataset were involved in this analysis. The expression of UBE2C in PCa was significantly higher than in benign prostate tissue (Figure 2A). Additionally, the difference in protein levels of UBE2C between PCa and normal prostate tissues was further confirmed in our PCa cohort. Figure 2B shows different levels of UBE2C protein expression in the prostatic tissues measured by immunohistochemistry staining. The immunohistochemical characteristics of UBE2C expression in the PCa tissues and the adjacent tissues were investigated using a paired Wilcoxon test, as displayed in Figure 2C. The paired Wilcoxon test shows that the UBE2C expression in PCa tissues was notably higher than in the adjacent tissues (*p* = 0.0002446). These results indicate that UBE2C is associated with tumorigenesis in PCa (*p* < 0.001, Figure 2C).

More importantly, in the TCGA cohort, we observed patients with *TP53* mutations have higher UBE2C expression levels (Supplementary Figure S2), and the highest UBE2C levels were observed in the T4 stage of PCa (Figure 2D). The levels of UBE2C also increased as the Gleason scores increased in PCa (Figure 2E). Additionally, in our cohort, Spearman’s rank correlation analysis was conducted to display the relationship between the protein level of UBE2C in PCa tissues and age, metastasis status, and Gleason score (Table 1). We found that PCa cell detection at the excision margin is significantly associated with UBE2C protein expression in the PCa tissues (r = 0.43, *p* = 0.044). This suggests the clinical significance of the UBE2C gene concerning surgical treatment. Figure 2F displays the Spearman correlation among clinical characteristics of PCa patients and UBE2C protein expression. As seen in Figure 2F, Gleason score (0–12) is the sum of the primary Gleason score (0–6) and secondary Gleason score (0–6); metastasis denotes whether the metastasis is found in lymph capillaries, capsula prostatica, seminal vesicle, or nerve; intensity denotes the detected UBE2C expression signal intensity on the microarray; area is the detected signal area on the microarray; expression is the multiple of the signal intensity and area. The statistical results show that the UBE2C protein expression in the cancerous tissues significantly correlates with the primary Gleason score (r = 0.51, *p* = 0.008). These results demonstrate that the expression level of UBE2C is strongly associated with PCa malignancy and that UBE2C is likely to be a biomarker for PCa, guiding surgery plans.

### 2.3. UBE2C Is an Independent Prognostic Biomarker in PCa

**Figure 3:**
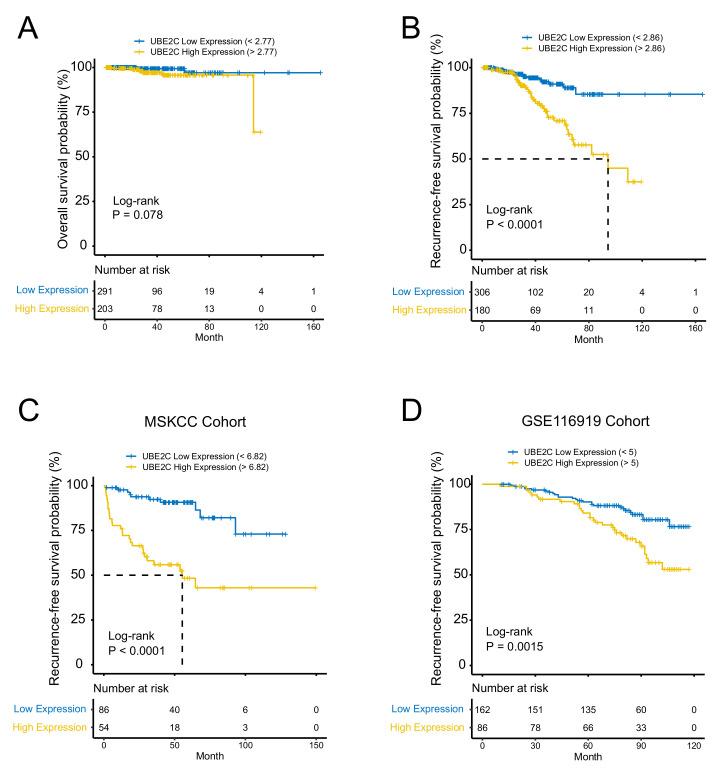
UBE2C is an independent prognostic biomarker in PCa. (**A**) No difference in overall survival between highly expressed UBE2C and low-expressed UBE2C groups. (**B**) Patients with low UBE2C levels had a significant recurrence-free (RFS) survival benefit compared to those with high UBE2C expression. (**C**) RFS confirmed in the MSKCC cohort. (**D**) RFS confirmed in GSE116918 cohort.

Given the level of UBE2C was found to be remarkably associated with grade and Gleason score in PCa, we then explored whether UBE2C was a prognostic biomarker for patients with PCa. Kaplan–Meier curves showed that under the optimal cutoff point, there was no difference in overall survival between highly expressed UBE2C and low-expressed UBE2C groups, while patients with low UBE2C levels had a significant recurrence-free survival benefit as compared with those with high UBE2C expression (Figure 3A,B). Furthermore, a univariable Cox proportion hazard regression model also identified UBE2C as a prognostic biomarker for PCa patients. Cox univariate regression analysis suggests that the pathological T stage, UBE2C expression, and Gleason Score were correlation factors of PCa prognosis (Table 2). The Cox multivariate regression analysis suggests pathological T stage and UBE2C expression were independent correlation factors of PCa prognosis (Table 2). In multivariate analysis, the Cox hazard ratio shows that at a given instant in time, someone with high UBE2C protein expression is 2.367 times as likely to die as someone with low UBE2C protein expression (*p* < 0.001, 95% CI: (1.138,4.923)) in the TCGA-PRAD database. Additionally, the predictive prognosis role of UBE2C on PCa recurrence-free survival has been further confirmed in two other PCa cohorts (Figure 3C,D).

## 3. Discussion

PCa is a high long-term survival disease when it is localized. More than any other cancer, PCa screening with the prostate-specific antigen (PSA) test increases the risk a man will have of facing a diagnosis of PCa [9]. However, metastatic PCa remains largely incurable even after intensive multimodal therapy [10]. It is quite meaningful to detect new molecular biomarkers highly related to the pathogenesis and metastasis of PCa with high specificity and sensitivity.

Our study explored hub genes in PCa progression and invasion, identified 17 distinct gene modules, and showed that the C2 cluster genes in the purple module were enriched in the cell cycle, *P53* signaling pathway, and DNA binding, bending functions, and pathways. After screening, we found that the UBE2C gene with high module membership in the purple module has higher gene significance scores for Gleason score and pathologic T stage.

UBE2C, as a crucial member of the ubiquitin-conjugating enzyme family (E2), plays a pivotal role in the ubiquitin–proteasome proteolytic (UPP) pathway. Disorders in the UPP pathway originate from the abnormal degradation of proteins encoded by some oncogenes and tumor suppressor genes, subsequently leading to the abnormal accumulation of these proteins in the body. Therefore, the UPP system is closely related to the occurrence and progression of cancers [11]. Emerging evidence has suggested that UBE2C was highly expressed in various tumors and acted as an oncogene, including ovarian cancer, non-small-cell lung cancer, cervical cancer, head and neck squamous cell carcinoma, etc. [12,13,14,15]. However, the exact function and molecular basis of UBE2C in PCa have remained elusive. Few studies have revealed the mechanisms of UBE2C in PCa. Our previous study validated the role of UBE2C in PCa progression in the human PCa cell lines LNCaP and PC-3. PCa cell proliferation rates were significantly slower than those of the control groups after UBE2C knockdown. Meanwhile, we observed that significantly fewer cells invaded the lower surface of the membrane through Matrigel after UBE2C knockdown. These results indicated that UBE2C was the critical factor in the proliferation and invasion of PCa cells [16]. Wang et al. demonstrated that UBE2C is a G1/S cell-cycle inhibitor-779 (CCI-779), an mTOR inhibitor, and inhibits UBE2C mRNA and protein expression in AR-positive CRPC cell models abl and C4-2B, in addition to its ability to block cell-cycle G1/S transition. Liu et al.’s study showed UBE2C inhibited the growth of melanoma cells via deactivating ERK/Akt signaling pathways and blocked G2/M transition through the downregulation of both the level and the activity of the mitosis-promoting factor (MPF), triggering the apoptosis of melanoma cells [8,17].

In this study, we continued to validate the public database and our cohort and found that UBE2C was strongly associated with malignant levels of PCa. Additionally, patients in the C2 cluster have more *TP53* mutations. Somatic *TP53* mutations occur in almost every type of cancer at rates from 38% to 50% in ovarian, esophageal, colorectal, head and neck, larynx, and lung cancers to about 5% in primary leukemia sarcoma, testicular cancer, malignant melanoma, and cervical cancer [18]. There was a significant association between the number of inactivated alleles and mRNA levels of *PTEN*, *TP53*, *CDKN1B*, *RB1*, and *CHD1* [19]. In metastatic PCa patients, the *TP53*, *PTEN*, and *RB1* tumor suppressor genes (TSGs) are recurrently altered in treatment-resistant PCa. The cooperative loss of two or more TSGs may drive more aggressive disease [20]. These studies may address why patients with UBE2C overexpression had a worse prognosis associated with *TP53* mutations.

Furthermore, the Cox hazard ratio shows that at a given instant in time, someone with high UBE2C protein expression is 3.29 times as likely to die as someone with low UBE2C protein expression in the TCGA-PRAD database. Additionally, the predictive prognosis role of UBE2C in PCa recurrence-free survival has been further confirmed in PCa.

However, there are some limitations to this study. Firstly, we measured UBE2C expression but did not test its related gene expressions such as *AR-V7* or *TP53*, which are important in normal prostate homeostasis [21]. Secondly, we did not include the benign prostate hyperplasia (BPH) tissue as UBE2C was also reported to be expressed in it and to be a critical factor in pathogenesis [22]. Lastly, due to the limited sample size, other variations by race, ethnicity, and geography need to be evaluated as they may have a different genetic map of PCa [23,24]. The underlying mechanisms deserve further study.

## 4. Materials and Methods

### 4.1. Weighted Correlation Network Analysis

WGCNA is an algorithm used in gene co-expression network identification by high-throughput expression profile mRNAs with different traits [25]: 1. By calculating the correlations of the top 8269 variation genes (the top 8269 genes with the largest variance between samples), a matrix of similarity was constructed. 2. By using the pickSoftThreshold function in the R WGCNA package, an appropriate soft-thresholding power β was selected. Then, this soft-thresholding power was used to increase the co-expression similarity and achieve scale-free topology. 3. The adjacency was transformed into a topological overlap matrix (TOM) using TOM similarity. 4. The corresponding dissimilarity (dissTOM) was also calculated. 5. By using dynamic tree cutting methods, co-expression gene modules were identified with the following major parameters: (1) maxBlockSize of 20,000 (2) minModuleSize of 30 and (3) deepSplit of two. The module eigengene (M.E.), which was the first principal component (P.C.) of each module’s gene expression matrix, was obtained by WGCNA to represent the expression profiles of module genes [26]. Highly similar modules with a height of M.E. in the clustering lower than 0.25 were merged. A clustering dendrogram was used to display the results of dynamic tree cutting and merging.

### 4.2. Hub Gene Identification

In WGCNA analysis for each gene expression profile, the gene significance (G.S.) was calculated as the absolute value of the correlation between the expression profile and each external trait (Gleason score, biochemical recurrence, and pathologic T). The module membership (MM) was defined as the correlation between the expression profile and each module eigengene. Module hub genes are highly connected intra-modular genes with the highest MM scores to the respective module [27]. The MM of each gene was calculated by WGCNA function signedKME, which correlates the expression profile of a gene with the M.E. of a module, so it quantifies how close a gene is to a given module. By calculating G.S. and MM values, highly significant genes for interesting traits and those that have high M.M.s in the interested module can be identified as hub genes. Finding hub genes is widely used in WGCNA analysis [28,29,30]. We also used the top 600 highly connected gene pairs (connect weight > 0.297) to construct a gene–gene interaction network. The network was displayed using Cytoscape software (3.7.0).

We followed data processing steps as outlined in the Horvath Lab UCLA protocol https://horvath.genetics.ucla.edu/html/CoexpressionNetwork/Rpackages/WGCNA/Tutorials/ (accessed on 20 March 2020).

### 4.3. Non-Negative Matrix Factorization

In the TCGA-PRAD cohort, transcriptome profiling data were virtually microdissected by employing the unsupervised non-negative matrix factorization (NMF) method as previously described [31] through the GenePattern module NMF [11]. The NMF algorithm, which is suitable for decomposing biological data, can factorize the gene expression matrix V (n genes × m samples) into two matrices: a gene factor matrix W of (n genes × k factors) and a sample factor matrix H of (m samples × k factors) [32]. Then, three distinct subtypes were dichotomized by the GenePattern module NMFConsensus using the gene expression of the genes in the WGCNA purple module.

### 4.4. Patient Cohort

The study sample, consisting of 90 patients with PCa, was obtained from a tissue specimen bank within Shanghai TUFEI Biotech Co., Ltd. All the patients were pathologically diagnosed with PCa and had been treated with prostatic surgery. Each study specimen was provided with cancerous tissue and adjacent tissue, which was 1.5 cm distanced from the cancerous margin. The clinical characteristics of 90 prostate patients are shown in Table 1.

### 4.5. Tissue Chip Production

Shanghai TUFEI Biotech Co., Ltd. (Shanghai, China) produced the tissue chip. Nine slices were collected for each specimen (three cancerous and three adjacent). Each was cut into 5 × 15 × 15 mm. Routine pathological hematoxylin–eosin (HE) staining was conducted on the wax blocks. Two pathologists performed cross-check diagnosis, and the typical pathological results were tagged on HE slides. A tissue chip production apparatus (Beecher Instruments, Inc., Tartu, Estonia) in the wax block was used to punch holes 1.5 mm in diameter. According to the tag position on the HE slices, the corresponding tissue was obtained from donor tissue wax blocks. Then, the target tissue chip was put into the array aperture of receptor wax blocks using a constant thermometer. Finally, the tissue chip of PCa tissue and matched adjacent tissue containing 180 microarray block points (HProA180PG04) was manufactured.

### 4.6. Immunohistochemistry Staining

The immunohistochemical experiment was performed using a two-step method. We put the chip into an automatic stain machine to remove the wax and rinsed it with nuclease-free water. We used high-temperature, high-pressure antigen retrieval, blocked the chip with an endogenous superoxide enzyme blocker for 10 min, and rinsed it with PBS. The first UBE2C antibody (1:500, 14682-1-AP, Proteintech Co., Ltd., Sankt Leon-Rot, Germany) was added to the chip, and the chip was placed overnight under 4 °C. Then, the second antibody EnVision™+/HRP (rabbit anti-rabbit immunoglobulin (DAKO) labeled by HRP) was added to the chip for 30 min, which was then rinsed with PBS 3 times, followed by DAB staining (DAKO) for 5 min. Then, the chip was rinsed with purified water, re-stained with hematoxylin, and put into an automatic dehydration machine, transparentized, and mounted.

### 4.7. Evaluation of Immunohistochemical Staining

Quantitative judgment: (1) Scoring methods of the staining intensity: at the low power field, we evaluated the tissues entirely as weakly positive, medium positive, and strongly positive; the staining color of weakly positive was defined to be light yellow (1 or +), medium positive as brownish-yellow (2 or ++), and strongly positive as brown (3 or +++). (2) Scoring methods of the positive staining rate: we selected three different staining intensity areas under the high-power lens and counted positive cells’ percentage among 100 cells for each area (X1%, X2%, and X3%) and calculated the average of the positive cells’ percentages as the overall positive staining rate.

We calculated tissue staining scores by multiplying the staining intensity score and the positive staining rate score.

### 4.8. Statistical Analysis

The expression of UBE2C genes in PCa and adjacent cancer tissues was compared using a paired Wilcoxon test. The association between the clinical characteristics of PCa patients and UBE2C protein expression was compared using Spearman’s correlation test. Based on the median of the UBE2C protein expression, we divided patients into high and low UBE2C expressions. The significance between the characteristic clinical parameters of PCa and UBE2C protein expression was tested using logistic regression with the following independent variables: age, primary and secondary Gleason Score, cancer cells detected on the excision margin (y/n), and metastasis. Log-rank test Kaplan–Meier curve, smooth H.R. curves, and Cox regression for survival analysis were performed using R package survival and smoothHR. The survival of patients belonging to different groups was compared using the Kaplan–Meier method, with the *p*-value determined by the log-rank (Mantel–Cox) test. A *p*-value less than 0.05 (two-tailed) was considered statistically significant. All statistical tests were performed using R software 3.6.1.

## 5. Conclusions

UBE2C is potentially a biomarker for PCa, and the overexpression of UBE2C is associated with worsened prognosis and *TP53* mutations.

## Figures and Tables

**Table 1:** Expression of UBE2C in relation to pathological and clinical variables.

Variables Studied	No. of Cases	Cancerous Tissues		*r* ^2^	*p*	Paracancerous Tissues		*r* ^2^	*p*
		Low	High			Low	High		
AGE (YEAR-OLD)				0.059	0.403			0.080	0.558
≤65	27 (30%)	19	8			24	3		
>65	63 (70%)	30	33			44	19		
PRIMARY GLEASON SCORE				0.268	0.008			−0.061	0.249
≤3	50 (55.7%)	29	21			35	15		
>3	40 (44.4%)	20	20			33	7		
SECONDARY GLEASON SCORE				0.102	0.621			0.024	0.534
≤3	45 (50%)	26	19			32	13		
>3	45 (50%)	23	22			36	9		
EXCISION MARGIN				0.187	0.044			0.047	0.507
+	6 (6.7%)	1	5			5	1		
-	84 (93.3%)	48	36			63	21		
METASTASIS				0.126	0.318			−0.220	0.719
+	22 (24.4%)	12	10			19	3		
-	68 (75.6%)	37	21			49	19		

$r^{2}$: correlation coefficient. The positive $r^{2}$ means positive correlation, while the negative $r^{2}$ is indicative of negative correlation. The closer the absolute value of $r^{2}$ is to 1, the greater the relativity.

**Table 2:** Summary of univariate and multivariate analysis for predicting PSA recurrence.

	Univariate		Multivariate	
Variables	HR (95%CI)	*p*-Value	HR (95%CI)	*p*-Value
Age (Continuous)	1.037 (0.990–1.087)	0.127	1.017 (0.971–1.066)	0.47
Pathological T stage (ref: ≤PT2c)	3.945 (2.076–7.498)	<0.001	2.635 (1.321–5.255)	0.006
Gleason Score (ref: ≤8)	2.971 (1.618–5.456)	<0.001	1.468 (0.707–3.050)	0.303
UBE2C (ref: <2.86)	3.29 (1.83–5.94)	<0.001	2.367 (1.138–4.923)	0.021

## Data Availability

The TCGA PCa cohort, composed of data from 52 benign and 495 PCa patients’ RNA-seq raw count and fragments trans per million (TPM), was obtained from the TCGA provisional database. Microarray data containing 248 PCa samples with biochemical recurrent information were obtained from GSE116918. The microarray data from the MSKCC cohort were obtained from http://cbio.mskcc.org/cancergenomics/prostate/data/ (accessed on 23 February 2021). This accompanies our tissue chip and patients’ cohort raw data at https://doi.org/10.7910/DVN/GEITQS.

## Supplementary Materials

The following supporting information can be downloaded at: https://www.mdpi.com/article/10.3390/ijms232213873/s1.

## Abbreviations

UBE2C	ubiquitin-conjugating enzyme 2C
PCa	prostate cancer
AR	androgen receptor
ADT	androgen-deprivation therapy
mCRPC	metastatic castration-resistant prostate cancer
NMF	non-negative matrix factorization
RFS	recurrence-free survival
UPP	ubiquitin–proteasome proteolytic
