# Supplementary figures and images for: Overexpression of Ubiquitin-Conjugating Enzyme E2C Is Associated with Worsened Prognosis in Prostate Cancer
Source: Int J Mol Sci. 2022 Nov 10;23(22):13873. doi: 10.3390/ijms232213873 (PMC9695011; doi:10.3390/ijms232213873)

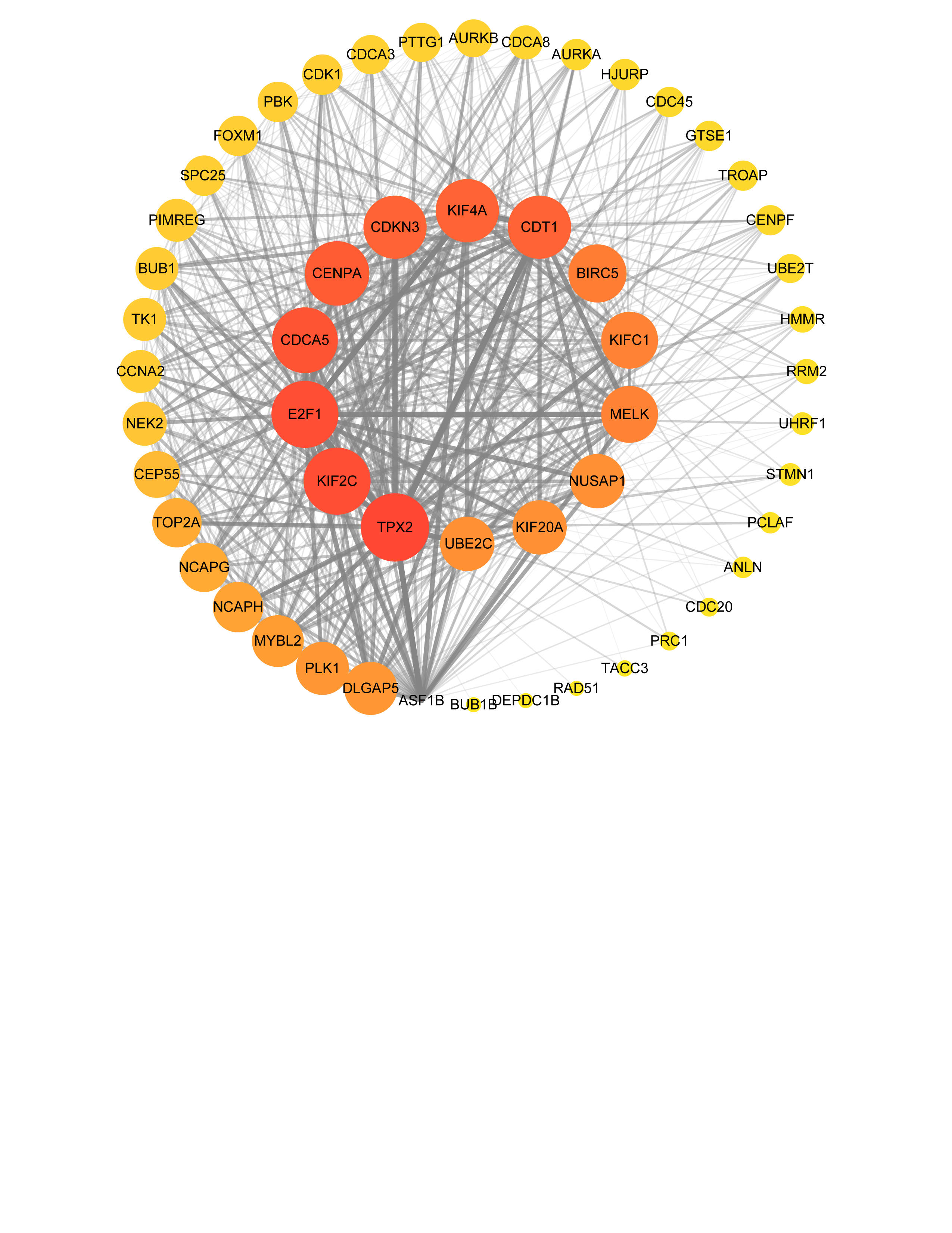

Supplement: Supplementary file 1 [file ijms-23-13873-s001.zip › Figure S1.tif]

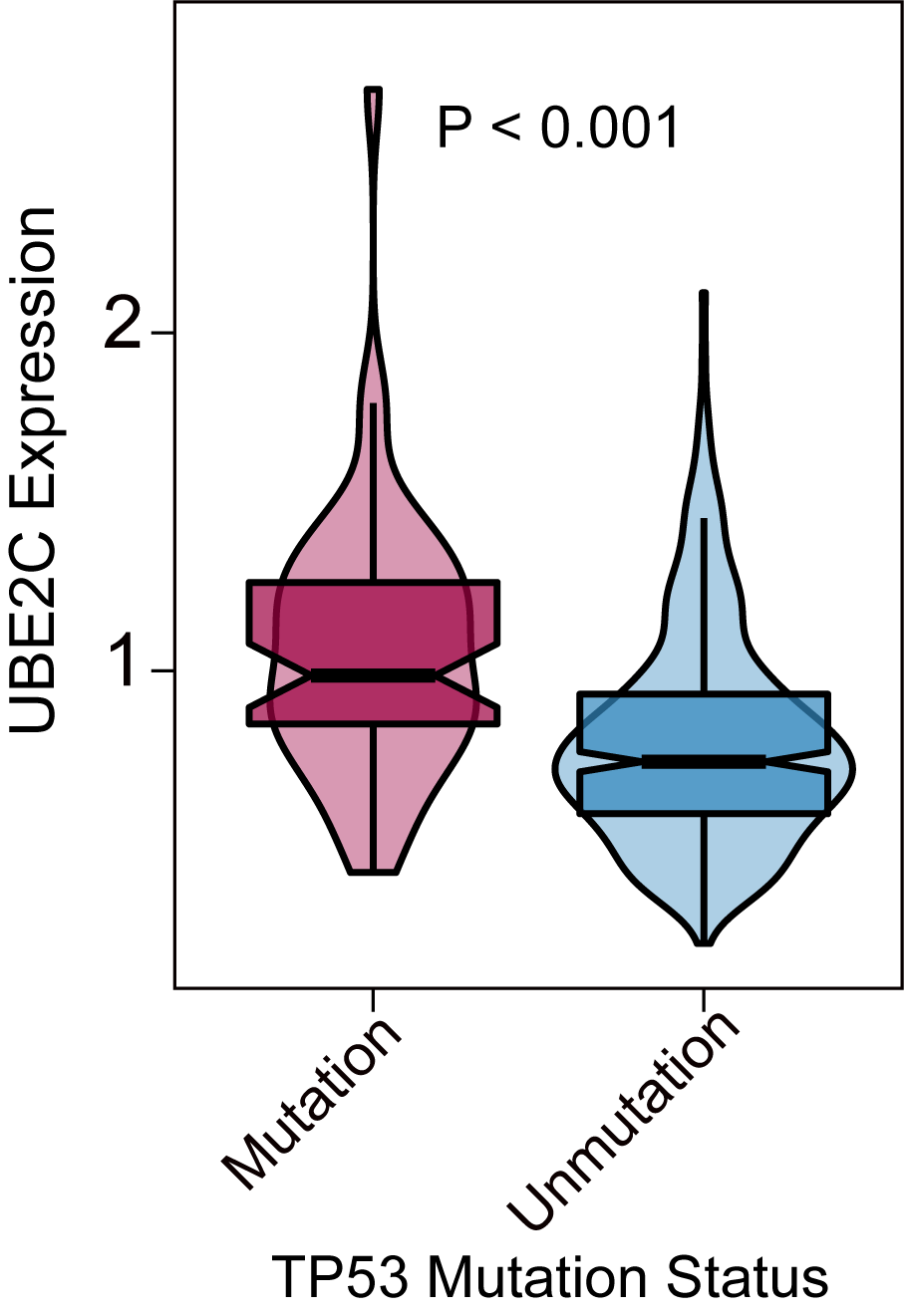

Supplement: Supplementary file 1 [file ijms-23-13873-s001.zip › Figure S2.tif]
